# Combined Area of Left and Right Atria May Outperform Atrial Volumes as a Predictor of Recurrences after Ablation in Patients with Persistent Atrial Fibrillation—A Pilot Study

**DOI:** 10.3390/medicina60010151

**Published:** 2024-01-13

**Authors:** Andrei D. Mărgulescu, Caterina Mas-Lladó, Susanna Prat-Gonzàlez, Rosario Jesus Perea, Roger Borras, Eva Benito, Francisco Alarcón, Eduard Guasch, Jose María Tolosana, Elena Arbelo, Marta Sitges, Josep Brugada, Lluís Mont

**Affiliations:** 1 Regional Cardiac Centre, Morriston Hospital, Swansea SA6 6NL, UK; 2 Unitat de Fibril·lació Auricular (UFA), Hospital Clinic, Universitat de Barcelona, 08036 Barcelona, Spain; cmasllad@gmail.com (C.M.-L.); lmont@clinic.cat (L.M.); 3 Institut d’Investigació Biomèdica August Pi i Sunyer (IDIBAPS), 08036 Barcelona, Spain; 4 Centro de Investigación Biomédica en Red (CIBER Cardiovascular), 08036 Barcelona, Spain

**Keywords:** atrial fibrillation, right atrium, left atrium, paroxysmal, persistent, remodelling

## Abstract

*Background and Objectives*: Left atrial (LA) remodelling and dilatation predicts atrial fibrillation (AF) recurrences after catheter ablation. However, whether right atrial (RA) remodelling and dilatation predicts AF recurrences after ablation has not been fully evaluated. *Materials and Methods*: This is an observational study of 85 consecutive patients (aged 57 ± 9 years; 70 [82%] men) who underwent cardiac magnetic resonance before first catheter ablation for AF (40 [47.1%] persistent AF). Four-chamber cine-sequence was selected to measure LA and RA area, and ventricular end-systolic image phase to obtain atrial 3D volumes. The effect of different variables on event-free survival was investigated using the Cox proportional hazards model. *Results*: In patients with persistent AF, combined LA and RA area indexed to body surface area (AILA + RA) predicted AF recurrences (HR = 1.08, 95% CI 1.00–1.17, *p* = 0.048). An AILA + RA cut-off value of 26.7 cm^2^/m^2^ had 72% sensitivity and 73% specificity for predicting recurrences in patients with persistent AF. In this group, 65% of patients with AILA + RA > 26.7 cm^2^/m^2^ experienced AF recurrence within 2 years of follow-up (median follow-up 11 months), compared to 25% of patients with AILA + RA ≤ 26.7 cm^2^/m^2^ (HR 4.28, 95% CI 1.50–12.22; *p* = 0.007). Indices of LA and RA dilatation did not predict AF recurrences in patients with paroxysmal AF. Atrial 3D volumes did not predict AF recurrences after ablation. *Conclusions*: In this pilot study, the simple measurement of AILA + RA may predict recurrences after ablation of persistent AF, and may outperform measurements of atrial volumes. In paroxysmal AF, atrial dilatation did not predict recurrences. Further studies on the role of RA and LA remodelling are needed.

## 1. Introduction

Atrial fibrillation (AF) is the most frequent sustained cardiac arrhythmia, with a prevalence that increases with age and can reach 6% in patients aged > 65 years [1]. The presence of AF is associated with decreased quality of life and increased risk of stroke, congestive heart failure, and mortality [1]. For these reasons, treatment of this arrhythmia is essential, and catheter ablation has become an established treatment for symptomatic AF refractory or intolerant to antiarrhythmic medication [2,3].

However, AF ablation is not without risk, some of which may be life threatening (e.g., cardiac tamponade, stroke, and atrial-oesophageal fistula). Thus, it is essential to evaluate pre-procedural probability of success in order to predict a satisfactory risk/benefit ratio. Several predictive factors for post-ablation AF recurrence have been identified: AF type (persistent vs. paroxysmal), extra cardiac factors (endurance training, hypertension, mitral valve disease, obstructive sleep apnoea [OSA], obesity) [4,5,6], and factors related to LA remodelling (LA dilatation, increased LA sphericity [7,8], scar burden [9], and increased LA pressure) [10]. Few studies have assessed the role of right atrial (RA) remodelling in predicting AF recurrences after ablation, and these studies, when performed, showed contradictory results [11,12,13,14,15,16,17,18]. In these studies, LA and RA remodelling has been assessed mainly using standard transthoracic echocardiogram or cardiac computed tomography. Cardiac magnetic resonance (CMR) imaging provides an alternative robust and reproducible modality to accurately assess atrial structure and remodelling [19].

The aim of the present study was to clarify the relationship between RA anatomical remodelling as determined by CMR and AF recurrence after AF ablation procedures.

## 2. Materials and Methods

### 2.1. Study Population

This is an observational study on a cohort of 85 consecutive patients with symptomatic, drug-refractory AF (≥1 antiarrhythmic), referred for first catheter ablation of AF, in whom a CMR study was obtained before the procedure. Data were obtained from a prospectively collected registry at the arrhythmia unit of our centre that includes clinical, ECG, echocardiographic, and CMR data. Exclusion criteria were: age < 18 or >75 years, presence of LA thrombus on trans-oesophageal echocardiography, presence of a mechanical prosthetic heart valve, claustrophobia, severe renal failure (glomerular filtration rate < 30 mL/min), gadolinium allergy, poor quality of CMR, implantable devices, pregnancy, or lactation.

Demographic data included type of AF (paroxysmal or persistent), history of OSA, endurance training, echocardiographically determined left ventricular ejection fraction, and left ventricular end-diastolic diameter. Definition of paroxysmal and persistent AF followed current recommendations [2,3].

Patients were included after written informed consent was obtained. The study protocol was reviewed and approved by the Hospital Clinic Barcelona research ethics committee (HCB/2018/0382).

### 2.2. Image Acquisition

Patients were screened for AF on an ambulatory basis on the day the CMR examination was to be carried out. If AF was documented, electric cardioversion was performed, following standard precautions regarding anticoagulant treatment and risk of stroke.

All CMR tests were performed in sinus rhythm with a 3-T system (MAGNETOM Trio™, a Tim^®®^ System, Siemens Healthcare, Erlangen, Germany) using electrocardiographic triggering and a 32-channel phased-array cardiovascular coil.

Short-axis cine images encompassing the ventricles and long axis (2-, 3- and 4-chamber views) were acquired using conventional steady-state free-precession sequence, during end-expiratory apnoea, using retrospective gating (repetition time 3.1 ms; echo time 1.4 ms; flip angle 50°; section thickness 6 mm; matrix 208 × 184; field of view 380 × 360 mm^2^; bandwidth 1335 Hz/px; parallel processing factor (GRAPPA) 2; and number of phases 30). After 25 min of intravenous administration of 0.2 mmol/kg of gadobutrol (Gadovist^®^, Bayer Hispania, Barcelona, Spain), a 3D delayed-enhancement sequence (3D DE) using free-breathing 3D navigator and an electrocardiographically gated inversion-recovery gradient-echo sequence was applied in the axial orientation. The acquired voxel size was 1.25 × 1.25 × 2.5 mm. A TI scout sequence was used to nullify the left ventricular myocardial signal and determine optimal TI. Patients were instructed to maintain steady, shallow breathing during image acquisition.

### 2.3. Image Analysis

**Figure 1:**
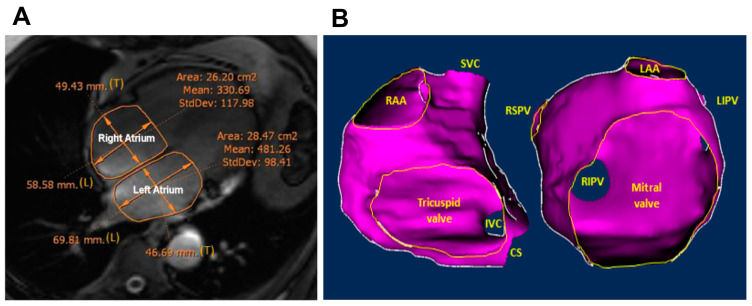
(**A**) Four-chamber view in the ventricular end-systolic phase, cine-sequence cardiac magnetic resonance showing how the longitudinal diameter (L), transversal diameter (T), and area were traced; (**B**) 3D reconstruction of the right and left atria (left anterior oblique view). The volumes were calculated after exclusion of the mitral/tricuspid valves, cava/pulmonary veins, and appendages.

Measurements were performed manually by 2 investigators, blinded to all clinical data. Four-chamber cine-sequences were selected at the ventricular end-systolic image phase to obtain the maximal atrial sizes. The diameters of both atria were measured from the posterior wall of the RA and LA to the centre of the tricuspid and mitral plane, respectively. The transversal diameters were measured perpendicular to the midpoint of the longitudinal diameters. RA and LA areas were measured by planimetry in the four-chamber view by manually tracking the wall contour of the atria. Pulmonary veins (PV) and RA and LA appendages were excluded (Figure 1).

To measure LA and RA volumes, a 3D DE sequence was used, tracking the LA and RA walls manually in each slice with the ADAS-AF software package (ADAS-AF, Galgo Medical, Barcelona, Spain), which reconstructs the 3D shape of each atrium. The investigator was then able to verify the correctness of the 3D shape and adjust the wall tracking as necessary. After exclusion of the PVs, LA appendage, and mitral valve, the LA volume was calculated. The RA volume was calculated after exclusion of superior and inferior vena cavas, coronary sinus, RA appendage, and the tricuspid valve. The LA and RA areas and volumes were indexed to the patients’ body surface area (AI_LA_ and AI_RA_, respectively).

### 2.4. Ablation Procedure

Catheters were introduced percutaneously through the right femoral vein; to access the LA, transseptal punctures were performed. Heparin was administered in order to maintain an activated clotting time > 250 s. AF ablation procedures were performed using either a cryoablation 28 mm balloon platform (Artic Front, Medtronic, Inc., Minneapolis, MI, USA; n = 12) or radiofrequency (RF) point-by-point ablation platform with an electroanatomic mapping system (CARTO, Biosense-Webster Waterloo, Belgium; n = 64 or NAVX, St. Jude Corporation, St. Paul, MI, USA; n = 9). Cryoablation was performed in 240 s deliveries at each PV ostia. When an electroanatomical mapping system was used, the CMR images were integrated into the navigation system to improve LA anatomic reconstruction, using at least 4 predefined PV ostia sites as landmark points and more than 20 other anatomic points to perform surface registration. Continuous RF lesions surrounding each ipsilateral PV were delivered. For both cryoablation and RF ablation procedures, the aim was complete isolation of all PVs, checking for bi-directional (entrance/exit) conduction block using pacing and a circular mapping catheter at the end of the procedure. If AF persisted after PVI, electrical cardioversion to sinus rhythm was performed in order to check PV exit conduction block. In some patients undergoing RF ablation, additional ablation lines were deployed along the LA roof and LA posterior wall joining contralateral encircling lesions and along the mitral isthmus, according to the operator’s criteria and depending on the type of AF.

### 2.5. Follow-Up

Patients were seen at 3, 6, and 12 months after catheter ablation and whenever required due to symptoms. Before each visit, 48 h Holter monitoring was performed. Patients were also asked to communicate any documented episode of AF or symptoms suggestive of AF recurrence. All patients continued oral anticoagulation. All of them received antiarrhythmic drugs (flecainide in the absence of structural heart disease or amiodarone otherwise) at least during the first 4 weeks after catheter ablation in order to manage early recurrences. Catheter ablation was considered successful if no AF or flutter lasting more than 30 s was recorded during follow-up after a 3-month blanking period without antiarrhythmic treatment.

### 2.6. Reproducibility of MRI Measurements

CMR measurements were repeated in 10 random patients, by the same observer twice on different days (intra-observer) and then by a second observer (inter-observer). The CMR measurements had good intra- and inter-observer reproducibility (all intra-observer coefficients of variation < 6%; all inter-observer coefficients of variation < 15%).

### 2.7. Statistical Analysis

The continuous variables were presented as the mean value ± standard deviation for normally distributed values, and median and interquartile range (IQR) for non-normally distributed values. The categorical variables were expressed as total number and percentages. The correlations are reported using the Pearson correlation coefficient. The effect of different variables on event-free survival was investigated using the Cox proportional hazards model. The hazard ratio (HR) and 95% confidence interval (CI) were also calculated. We used receiver operating characteristic (ROC) methodology to evaluate the optimal cut-off value for predicting recurrence in our sample. The event-free survival of patients was evaluated with the Kaplan–Meier method. A two-sided type I error of 5% was used for all tests. Statistical analysis was performed using Statistical Package for Social Sciences (SPSS) version 23.0.

## 3. Results

Baseline characteristics of the study population are shown in Table 1. RA volumes were well correlated with LA volumes (r = 0.57, *p* < 0.001).

AF recurrences after ablation occurred in 35 patients (41.1%). The recurrence rate after ablation was similar in patients with paroxysmal AF, compared to persistent AF (38.8% vs. 45.0%, *p* = 0.52). Compared to patients with paroxysmal AF, patients with persistent AF had larger LA and RA indexed volumes (LA indexed volume, 51.4 ± 15.6 vs. 42.1 ± 9.3 mL/m^2^ and RA indexed volume, 58.6 ± 19.3 vs. 45.9 ± 10.7 mL/m^2^, respectively; LA + RA indexed volume, 110.2 ± 30.4 vs. 88.0 ± 16.6 mL/m^2^; *p* ≤ 0.001 for all). However, the AI_LA_ and AI_LA_ did not differ between groups (AI_LA_, 14.3 ± 3.4 vs. 13.0 ± 2.7 cm^2^/m^2^, *p* = 0.07; AI_RA_, 12.8 ± 3.0 vs. 12.0 ± 2.2 cm^2^/m^2^, *p* = 0.18; AI_LA+RA_, 27.1 ± 5.6 vs. 25.1 ± 4.1 cm^2^/m^2^, *p* = 0.06). Patients with AF recurrences had higher combined LA + RA volumes and areas, compared to patients without recurrences (Table 2). In univariate Cox regression analysis, only AI_LA_ and AI_LA+RA_ were associated with AF recurrences after ablation, regardless of clinical presentation (Table 3).

Both AI_RA_ and AI_LA+RA_ were predictors of AF recurrence in patients with persistent AF; no parameters were predictive in patients with paroxysmal AF (Table 2). Using the ROC analysis, the best cut-off value for AI_RA_ for predicting recurrence after ablation of persistent AF was 11.3 cm^2^/m^2^. This cut-off value had good sensitivity (77%) but poor specificity (57%), making it not clinically useful according to the Kaplan–Meier analysis (HR 1.56, 95% CI 0.55–4.38; *p* = 0.40).

**Figure 2:**
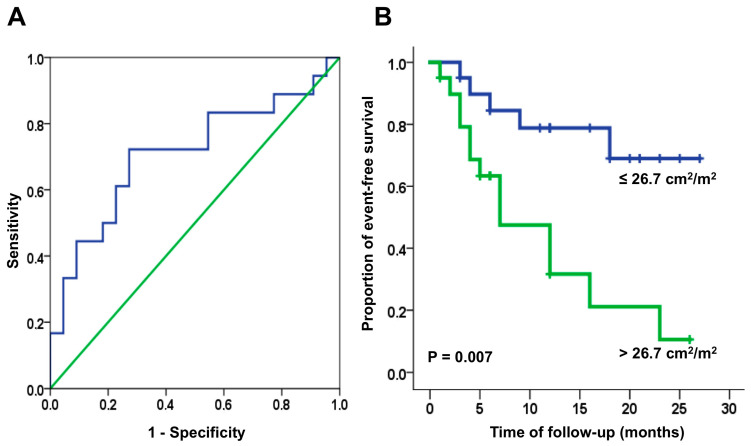
Receiver operating curve (**A**) and Kaplan–Meier recurrence-free survival curve (**B**) in patients with persistent AF, using an AI_LA+RA,_ cut-off value of 26.7 cm^2^/m^2^.

The best cut-off value AI_LA+RA_ for predicting recurrence after ablation of persistent AF was 26.7 cm^2^/m^2^ (sensitivity 73%, specificity 78%). The Kaplan–Meier recurrence-free survival curve using this cut-off value for AI_LA+RA_ shows widely divergent curves immediately after ablation (HR 4.28, 95% CI 1.50–12.22; *p* = 0.007) (Figure 2). Atrial volumes were not predictive of AF recurrences when analysed for all patients, and separately by AF clinical type.

## 4. Discussion

Our data suggest that AI_LA+RA_ may predict recurrences after ablation in patients with persistent AF (sensitivity 72%, specificity 73% for an AI_LA+RA_ cut-off value of 26.7 cm^2^/m^2^), and may outperform measurements of atrial volume. Atrial dilatation did not predict AF recurrences in patients with paroxysmal AF.

Prior studies have reported contradictory results regarding the ability of parameters of RA dilatation to predict AF recurrences after cardioversion or ablation. Akutsu et al. suggested that LA and RA volumes are predictors of 6-month recurrence in patients with paroxysmal AF [11]. Other studies that included mixed populations (two-thirds of which generally consisted of patients with paroxysmal AF) suggest that LA and RA indexed volumes predict AF recurrences in patients with persistent but not paroxysmal AF; under multivariable analysis, however, only LA indexed volume remained as an independent predictor of AF recurrence in patients with persistent AF [13]. In similar populations, RA indexed volume may predict early (<3 months) recurrences, while LA indexed volume may predict late (1 year) recurrences [15]. In patients with long-lasting persistent AF, RA dilatation [12] and the RA/LA volume ratio [16] have been suggested as independent predictors of AF recurrences after ablation. In a recent pooled analysis of both paroxysmal and persistent AF patients, we have shown that RA sphericity is associated with recurrences after AF ablation, while atrial areas, volumes, and scar burden (as detected by CMR) were not [17]. In persistent AF, RA indexed volume may predict AF recurrences after electrical cardioversion [14,18]. A summary of these studies is provided in Table 4.

The present results generally fit with these prior observations, but some differences merit discussion. In this cohort, no predictors of AF recurrence were found in patients with paroxysmal AF. This is in keeping with prior results and with the current understanding of the pathophysiology of paroxysmal AF, where triggers (as opposed to substrate alterations) are thought to play the dominant role. Some overlap in mechanisms is to be expected, as the clinical classification of AF types into paroxysmal and persistent is likely to be imprecise, and as many as 40% of patients with AF may be asymptomatic [20]. This incongruence between the putative AF mechanism and clinical AF type may explain the similar rates of AF recurrence in patients with paroxysmal versus persistent AF observed in our study. Moreover, studies in which LA and RA dilatation could predict AF recurrences in patients with paroxysmal AF likely included more patients with atrial substrate alterations than did studies which found no such relationships. For example, Akutsu et al. reported mean LA and RA volumes of 100 ± 32 mL and 83 ± 36 mL [11], compared with the values reported by Moon, 74 ± 19 mL and 82 ± 23 mL, respectively [13].

In patients with persistent AF, atrial anatomical substrate alterations may play the dominant role in maintaining AF. Dilatation and the amount of fibrosis (as detected by late gadolinium enhancement) have been shown to predict the risk of AF recurrence in these patients. However, triggers and AF maintenance substrates (e.g., rotors) have been found at the RA level in approximately 1/3 of patients undergoing AF ablation [21]. In this context, we provide further evidence that simple parameters of RA dilatation in patients who underwent pre-procedural CMR provide useful prognostic information in assessing the risk of AF recurrence in patients with persistent AF and that combined assessment of RA and LA dimensions may better identify the patients at risk of AF recurrence after ablation, compared with either parameter alone.

It is not clear why the simple measurement of cross-sectional atrial area identified this risk of AF recurrence after ablation more accurately than the more complex determination of 3D atrial volume in the present study; the most likely explanation is related to the small number of patients included in each group. However, atrial volumes were higher in patients with AF recurrences after ablation compared with patients without recurrences in the whole population (Table 2). It is also possible that the pattern of atrial dilatation (whether it develops predominantly in the transversal plane, longitudinal plane, or globally) may be important to the risk of AF recurrence after ablation [17]. These questions merit further studies.

### Study Limitations

This study has several limitations. The sample size was relatively small, especially regarding the subgroups of paroxysmal and persistent AF, and all analysis were not pre-specified, and no power calculation was performed. As a result, confirmation of our findings from future studies is required, as a type 2 error cannot be excluded. Also, the small sample size of our study is the most likely explanation why our study did not confirm previously published indices (clinical type of AF, hypertension, OSA, endurance training) that predict AF recurrence after AF ablation. It is also likely that patients with severe LA dilatation were not considered for ablation according to current practice, creating a reference bias towards lower atrial volumes. Lastly, whether measurement of LA and RA areas by echocardiography from the apical four-chamber view would provide similar predictive value as CMR measurements was not investigated in this study and should be further evaluated.

## 5. Conclusions

In this pilot study, the simple measurement of AI_LA+RA_ (cut-off value: 26.7 cm^2^/m^2^) may predict recurrences after ablation in patients with persistent AF, and may outperform measurements of atrial volumes. Indices of LA and RA dilatation do not predict AF recurrences in patients with paroxysmal AF.

## Figures and Tables

**Table 1:** Baseline characteristics.

Age, Years (Mean, SD)	56.9 ± 9.1
Male sex (n, %)	70 (82.4%)
Height, cm (mean, SD)	174.0 ± 9.6
Weight, kg (mean, SD)	84.2 ± 14.8
Persistent AF (n, %)	40 (47.1%)
Hypertension (n, %)	40 (47.1%)
OAS (n, %)	12 (14.1%)
Endurance training (n, %)	12 (14.3%)
LVEF, % (mean, SD)	59.3 ± 8.1
LV end-diastolic diameter, mm (mean, SD)	52.2 ± 5.0
Follow-up, months (median, IQR)	11.0 (12.0)
Recurrence (n, %)	35 (41.1%)

Note: SD, standard deviation; IQR, interquartile range; Persistent AF, long-standing persistent atrial fibrillation; OAS, obstructive apnoea syndrome; LVEF, left ventricular ejection fraction; LV, left ventricle.

**Table 2:** Clinical characteristics, echocardiographic data, procedure-related parameters, and CMR parameters in relation to atrial fibrillation recurrence after ablation.

	General			Paroxysmal AF			Persistent AF		
	No Recurrence	Recurrence	*p* Value	No Recurrence	Recurrence	*p* Value	No Recurrence	Recurrence	*p* Value
	N = 50	N = 35		N = 28	N = 17		N = 22	N = 18	
Clinical characteristics									
Age, years (mean, SD)	57.6 ± 9.6	55.9 ± 8.3	0.42	57.1 ± 11.0	57.5 ± 9.1	0.89	58.3 ± 7.8	54.2 ± 7.4	0.11
BSA, m^2^ (mean, SD)	2.00 ± 0.21	2.03 ± 0.23	0.93	1.96 ± 0.22	2.00 ± 0.23	0.60	2.05 ± 0.19	2.06 ± 0.22	0.94
Men, n (%)	41/50 (82.0%)	29/35 (82.9%)	1.00	22/28 (78.6%)	12/17 (70.6%)	0.72	19/22 (86.4%)	17/18 (94.4%)	0.61
Hypertension, n (%)	23/50 (46.0%)	17/35 (48.6%)	0.83	11/28 (39.3%)	7/17 (41.2%)	1.00	12/22 (54.6%)	10/18 (55.6%)	0.97
Diabetes, n (%)	3/41 (7.3%)	3/28 (10.7%)	0.68	1/24 (4.2%)	1/15 (6.7%)	1.00	2/17 (11.8%)	2/13 (15.4%)	1.00
OSA, n (%)	7/50 (14.0%)	5/35 (14.3%)	1.00	3/28 (10.7%)	3/17 (17.7%)	0.66	4/22 (18.2%)	2/18 (11.1%)	0.56
Endurance training, n (%)	8/50 (16.0%)	4/34 (11.8%)	0.75	5/28 (17.9%)	1/16 (6.3%)	0.39	3/22 (13.6%)	3/18 (16.7%)	1.00
Cardiomyopathy, n (%)	8/48 (16.7%)	6/32 (18.8%)	1.00	4/28 (14.3%)	1/16 (6.3%)	0.64	4/20 (20.0%)	5/16 (31.3%)	0.47
Echocardiographic data									
LVEF, % (median, IQR)	60.0 (5.0)	60.0 (0.0)	0.57	63.0 (5.0)	60.0 (5.0)	0.81	60.0 (10.5)	60.0 (4.0)	0.58
LVEDD, mm (mean, SD)	49.5 ± 5.7	51.5 ± 6.7	0.12	49.4 ± 5.3	51.7 ± 5.3	0.22	49.8 ± 6.4	51.3 ± 7.8	0.56
LA diameter, mm (mean, SD)	41.5 ± 5.4	43.3 ± 4.8	0.19	41.2 ± 6.2	42.5 ± 5.3	0.51	41.2 ± 4.4	44.1 ± 4.3	0.13
Procedure-related parameters									
Duration of procedure, hours (median, IQR)	180.0 (67.5)	200 (62.5)	0.93	180.0 (100.0)	200.0 (80.0)	0.46	195.0 (60.0)	187.5 (52.5)	0.95
Fluoroscopy time, min (median, IQR)	23.0 (17.8)	24.0 (11.5)	0.75	23.0 (16.5)	46.0 ± 75.2	0.25	23.4 ± 11.1	22.9 ± 9.7	0.88
CMR parameters									
AI_LA_, cm^2^/m^2^ (mean, SD)	13.0 (2.9)	14.5 (3.2)	0.021	12.6 (2.5)	13.8 (2.9)	0.16	13.4 (3.2)	15.3 (3.4)	0.094
AI_RA_, cm^2^/m^2^ (mean, SD)	11.9 (2.2)	13.0 (3.1)	0.07	12.0 (2.3)	12.0 (2.1)	0.98	11.8 (2.1)	14.0 (3.6)	0.026
AI_LA+RA_, cm^2^/m^2^ (mean, SD)	24.9 (4.2)	27.5 (5.6)	0.015	24.6 (4.1)	25.8 (4.1)	0.36	25.3 (4.4)	29.2 (6.3)	0.026
LA indexed volume (mL/m^2^) (mean, SD)	44.3 (12.1)	49.5 (14.8)	0.08	40.6 (8.4)	44.4 (10.5)	0.19	49.0 (14.4)	54.2 (16.9)	0.30
RA indexed volume (mL/m^2^) (mean, SD)	49.0 (13.0)	55.7 (20.0)	0.07	45.4 (10.0)	46.7 (11.9)	0.70	53.8 (15.0)	64.2 (22.5)	0.09
LA + RA indexed volume (mL/m^2^) (mean, SD)	93.4 (22.0)	105.2 (30.4)	0.043	86.1 (15.0)	91.1 (19.0)	0.33	103.2 (26.1)	118.5 (33.6)	0.12

Note: SD, standard deviation; IQR, interquartile range; BSA, body surface area; OSA, obstructive sleep apnoea; RA, right atrium; LA, left atrium; CMR, cardiac magnetic resonance imaging; LVEF, left ventricular ejection fraction; LVEDD, left ventricular end-diastolic diameter; AI_LA_, left atrium area indexed for body surface area; AI_RA,_ left atrium area indexed for body surface area; AI_LA+RA,_ combined left and right atria area indexed for body surface area.

**Table 3:** Univariate predictors of atrial fibrillation recurrence according to clinical atrial fibrillation type.

	General		Paroxysmal AF		Persistent AF	
	HR (95% CI)	*p* Value	HR (95% CI)	*p* Value	HR (95% CI)	*p* Value
AI_LA_ (cm^2^/m^2^)	1.14 (1.02–1.28)	0.021	1.19 (0.98–1.44)	0.09	1.13 (0.97–1.32)	0.13
AI_RA_ (cm^2^/m^2^)	1.12 (0.98–1.23)	0.09	0.96 (0.75–1.24)	0.78	1.16 (1.00–1.33)	0.043
AI_LA+RA_ (cm^2^/m^2^)	1.08 (1.01–1.16)	0.017	1.08 (0.94–1.25)	0.27	1.08 (1.00–1.17)	0.048
LA indexed volume (mL/m^2^)	1.02 (1.00–1.05)	0.11	1.04 (0.98–1.10)	0.19	1.02 (0.99–1.05)	0.30
RA indexed volume (mL/m^2^)	1.02 (1.00–1.03)	0.12	1.01 (0.96–1.06)	0.77	1.02 (0.99–1.04)	0.18
LA + RA indexed volume (mL/m^2^)	1.01 (1.00–1.02)	0.07	1.02 (0.99–1.05)	0.33	1.01 (1.00–1.03)	0.18

Note: RA, right atrium; LA, left atrium; AI_LA_, left atrium area indexed for body surface area; AI_RA,_ left atrium area indexed for body surface area; AI_LA+RA,_ combined left and right atria area indexed for body surface area. Bolded numbers denote significant predictors.

**Table 4:** Summary of main studies dealing with right atrial remodelling as a predictor of atrial fibrillation recurrence.

Study	N	Follow-Up	Type of AF	Intervention	Method	Parameter	Results
Shin 2008 [7]	68	6 months	59% paroxysmal	Ablation	2D echo	RAVi, LAVi	Univariate analysis: RAVi and LAVi predicted recurrences. Multivariate analysis: only LAVi predicted recurrences. Cut-off value 34 mL/m^2^: Ss 70%, Sp 91%.
Akutsu 2011 [11]	65	6 months	Paroxysmal	Ablation	CT angiogram	RAV, LAV	RAV correlated with LAV (r = 0.4, *p* < 0.01). RA volume [OR, 1.04; 95% confidence interval (CI), 1.02 to 1.07, *p* < 0.0001], - LA volume: OR 1.04 [95% CI, 1.01 to 1.06, *p* = 0.002] Multivariate: RA and LA volumes remained predictive of AF recurrence. Cut-off values: - RAV ≥ 87 mL: OR 13.4 (3.2–54.9); Ss 81.3%, Sp 75.5% - LAV ≥ 99 mL: OR 9.8 (2.4 –39.6); Ss 81.3%, Sp 69.4% - LAV + RAV: OR 46 (9–233.9); Ss 75%, Sp 93.9%
Zhao 2013 [12]	208	19.9 months (average)	Long-term persistent	Ablation	2D echo	RA enlargement (categorical value)	Patients with AF recurrence had higher rates of: - RA enlargement (67.7 vs. 45.4%, *p* = 0.018); - LA diameter (49.4 ± 6.2 vs. 46.5 ± 5.3 mm, *p* = 0.007). - ≥2 procedures (58.8% vs. 27.0%, *p* < 0.001); - longer AF duration (82.4 ± 44.8 vs. 50.8 ± 42.8 months, *p* < 0.001). In the multivariate analysis, RA enlargement, ≥2 procedures, and AF duration were independent predictors of AF recurrence.
Moon 2013 [13]	242	18 months (average)	66% paroxysmal	Ablation	CT angiogram	RAVi, LAVi	RAVi correlated with LAVi in both paroxysmal and persistent AF (r = 0.6; *p* < 0.01 for both) Overall: - LAVi independently predicted recurrences (10 mL/m^2^ increase; HR: 1.22, 95% CI: 1.09–1.36, P b 0.001) - RAVi did not predict recurrences independently. In subgroup analysis: - Paroxysmal AF: Neither RAVi nor LAVi predicted recurrences, despite borderline significance for RAVi (for each 10 mL/m^2^ increase; HR: 1.21, 95% CI: 1.00–1.48, *p* = 0.05). - Persistent AF: LAVi was an independent predictor for each 10 mL/m^2^ increase (HR: 1.19, 95% CI: 1.03–1.36).
Luong 2015 [14]	95	6 months	Persistent	Cardioversion	2D echo	RAVi, LAVi	RAVi was superior to LAVi in predicting recurrences: - RAVi cut-off 42 mL/m^2^: AUC 0.77, Ss 71%, Sp 85% - LAVi cut-off 48 mL/m^2^: AUC 0.64, Ss 53%, Sp 79%
Moon 2015 [15]	111	12 months	61% paroxysmal	Ablation	CT angiogram	RAVi, LAVi	- Early recurrence was independently associated with RAVi (for each 10 mL/m^2^ increase; odds ratio [OR], 1.31; 95% CI, 1.03–1.66, *p* = 0.03), but not with LAVi. - However, both RAVi and LAVi failed to predict 6-month outcomes independently. - LAVi was the only independent predictor of 1-year recurrence (for each 10 mL/m^2^ increase; OR, 1.36; 95% CI, 1.08–1.71, *p* = 0.009).
Sazaki 2016 [16]	70	15 months	Long-term persistent	Ablation	CT angiogram	RAVi, LAVi RA/LA volume ratio	RA (but not LA) dilatation are risk factors for AF recurrence after ablation (AUC 0.64; 95% CI, 0.49–0.79). RA/LA volume ratio is a better predictor of recurrences than RA volume alone: RA/LA volume ratio cut-off 100.1%; hazard ratio, 1.05; 95% CI, 1.00–1.10; *p* = 0.039; Ss 85.7%, Sp 71.4%, AUC 0.77, 95% CI, 0.66–0.88.
Gunturiz-Beltrán 2022 [17]	100	24 months (median)	55% paroxysmal	Ablation	CMR	RAVi, LAVi Sphericity index	Pooled results suggest only the sphericity index was predictive of AF recurrences after ablation, while RA and LA area and volumes were not. Results not split between paroxysmal and persistent AF.
Döring 2023 [18]	589	Immediate success	Persistent (86%) and long-term persistent (14%)	Cardioversion	2D echo	RAi area, LAVi	iRA area (OR, 0.27; 95% CI, 0.12–0.69; AUC 0.71) but not LAVi (OR, 1.16; 95% CI, 1.05–1.56) is a strong echocardiographic indicator of cardioversion success.

Note: BSA, body surface area; RA, right atrium; LA, left atrium; CMR, cardiac magnetic resonance imaging; LVEF, left ventricular ejection fraction; LVEDD, left ventricular end-diastolic diameter; RAVi, right atrial indexed volume; RAi area, right atrium indexed area; LAVi, left atrial indexed volume; Ss, sensitivity; Sp, specificity.

## Data Availability

Data available upon reasonable request.
